# Barriers and facilitators to the delivery of delirium care in intensive care units: an analysis informed by the Theoretical Domains Framework

**DOI:** 10.1111/anae.70017

**Published:** 2025-10-07

**Authors:** Roxanne M. Parslow, Ben Gibbison, Maria Pufulete, Kathryn M. Rowan, Andrew J. Moore, the OPTIC Study Group, Mike Grocott, Emma Hopkins, Alicia O'Cathain, Paul Moran, Claire Black, Cathrine McKenzie, Andy Gibson, Susie Robinson‐Molloy, Burak Kundakci, Andrew Booth, Katherine L. Jones, Sarah Smith, James Long, Molly Potter, A. Francis Johnson

**Affiliations:** ^1^ Bristol Medical School University of Bristol Bristol UK; ^2^ Department of Anaesthesia University Hospitals Bristol and Weston NHS Foundation Trust Bristol UK; ^3^ Intensive Care National Audit and Research Centre London UK

**Keywords:** delirium, intensive care unit, qualitative, theoretical domains framework

## Abstract

**Introduction:**

Delirium is common in patients who are critically ill and associated with increased mortality and long‐term cognitive impairment. The objective of this study was to explore the barriers and facilitators to the use of existing ICU delirium identification tools and implementation of care protocols.

**Methods:**

Semi‐structured interviews were conducted with healthcare professionals working in UK NHS ICUs. Purposive sampling was utilised to recruit a diverse range of participants to account for profession; user status of delirium identification tools or care packages; size of unit; and UK location. Thematic analysis was undertaken using the Theoretical Domains Framework.

**Results:**

Twenty‐one ICU healthcare professionals were interviewed from 20 hospitals. Participants included consultants (n = 9); nurses (n = 8); advanced critical care practitioners (n = 2); and allied health professionals (n = 2). Five major barriers to effective delirium care emerged: lack of prioritisation; lack of structured ICU delirium care protocols; inability to implement interventions due to physical space constraints and/or lack of resources; loss of experienced staff; and changes in ICU nursing culture. Facilitators included the presence of clear protocols; continued staff training; increased awareness; delirium champions; post‐ICU follow‐up clinics; family engagement; effective communication; and the use of digital prompts as a component of mandatory electronic documentation.

**Discussion:**

Although ICU delirium is acknowledged as being important by clinical staff, management is often hindered by systemic and cultural barriers. Healthcare professionals highlighted the need for protocol‐driven care, enhanced training and awareness, and the inclusion of families in care processes. These findings will inform the design of a multicomponent care package to improve delirium care in the ICU.

## Plain Language Summary

Delirium is a health issue where people become very confused or act strangely because of another health problem, such as an operation or an infection. It is common in intensive care units, where very ill people are treated. People who get delirium need to stay in hospital for longer. They may also have problems with thinking and memory after they leave hospital. This study was part of a project to make a new care plan to prevent and treat delirium in intensive care units. Researchers spoke with 21 doctors, nurses and other staff from 20 intensive care units in the UK. They asked about how staff spot delirium, prevent it and care for patients with it. The goal was to understand what helps and what gets in the way.

The study found five problems: some doctors and nurses don't see delirium as important; many ICU units don't have clear plans to how to deal with delirium; many units don't have enough staff; experienced staff are leaving; and nurses are now doing more technical tasks instead of traditional caring roles. But the study also found things that can help. For example, having a clear care plan for delirium, training staff often and making them aware of delirium, or having staff who just focus on delirium can help. Also, good communication, setting reminders for staff to check on patients, involving families in care and checking on patients after they go home. In short, most doctors and nurses know that delirium matters but they struggle to deal with it properly. To make things better, intensive care units need clear care plans, more training and more family involvement.

## Introduction

Delirium in the ICU is an acute confusional state that occurs in up to 70% of patients who are critically ill [1]. Over 200,000 patients are admitted to ICUs in England and Wales annually [2], creating a significant burden on patients, their families and healthcare providers. The diagnosis of ICU delirium will likely increase given trends to admit more older patients with multiple long‐term conditions. Delirium is associated with higher mortality, longer duration of hospital and ICU stays, and increased hospital costs [3]. This is also associated with post‐traumatic stress disorder [4] and longer‐term development of cognitive dysfunction and dementia [1, 5, 6].

Effective care for ICU delirium involves prevention, early recognition and focused management. Delirium identification tools (e.g. Confusion Assessment Method for ICU (CAM‐ICU)) [7] and the Intensive Care Delirium Screening Checklist (ICDSC) [8] can be used to detect and monitor symptoms. Implementation of structured care packages (such as the ABCDEF bundle) have been shown to reduce the incidence and duration of delirium [9]. However, these care packages are used inconsistently, both in the UK and internationally [10, 11]. Many of the components of ICU delirium care are non‐pharmacological: frequent reorientation; sleep promotion; early mobilisation; and minimal sedation. Pharmacological interventions such as antipsychotics have limited benefit [12].

The OPTIC study (OPTimising the prevention, identification and management of ICU Delirium) aims to explore current practice in prevention, identification and management of ICU delirium in the UK. Within the overarching project, this qualitative study was designed to understand current healthcare professional experience and behaviours in ICU delirium care to inform intervention development.

## Methods

The study protocol was approved by the University of Bristol Faculty of Health Sciences Research Ethics Committee. Following verbal consent, semi‐structured interviews were undertaken by RMP with healthcare practitioners involved in the care of patients with ICU delirium in the NHS. Participants that completed a survey to define ICU delirium practice for the OPTIC study through the Intensive Care National Audit and Research Centre (ICNARC) [13] and opted to be contacted for a qualitative interview were purposively sampled. We sought to understand how experiences of ICU delirium care differed across varying hospital contexts and for different clinical roles. Purposive maximum variation sampling was utilised [14]. The following dimensions were considered [15]: profession; hospital use of ICU delirium identification tools or care packages; hospital size and location. A topic guide (online Supporting Information Appendix S2) was developed that incorporated questions based on the Theoretical Domains Framework [16]. This is a synthesis of 33 psychological and organisational theories summarised in 14 domains to provide a guide towards implementing evidence‐based practice. This approach provides a theoretical framework and lens through which to view the cognitive, affective, social and environmental influences on healthcare practitioner behaviour [16]. The topic guide was developed using an iterative process involving investigators with qualitative expertise, clinical experts and patient representatives (The OPTIC Study group).

Questions were structured to explore identification tools and ICU delirium prevention and management practices. Prompts were provided to capture the range of constructs within each theoretical domain. Pilot interviews were conducted with four clinicians to iteratively develop the topic guide [16]. Interviews continued until thematic saturation was achieved where the relevant themes and Theoretical Domains Framework structure had been explored and described [15]. Interviews were audio‐recorded, transcribed verbatim and transcripts checked for accuracy and uploaded to NVivo qualitative data management software (Lumivero, Denver, CO, USA) for analysis.

Transcripts were inductively coded for content and meaning and used to generate themes that encapsulated barriers and facilitators to ICU delirium screening and management. Following this, deductive coding was undertaken using the 14‐domain Theoretical Domains Framework [17] and the inductive themes matched (online Supporting Information Table S1). The interviews and analysis were led by RMP, a white, female qualitative researcher, who brought experience of health services research but no previous background in the ICU setting (outsider role). Reflexive field notes, made after individual interviews, were referred to when developing the themes to enhance the transparency of the analysis. A senior qualitative researcher (AJM) double‐coded 10% of the transcripts and assigned the Theoretical Domains Framework categories. Overall themes were discussed, with final coding agreed to improve the trustworthiness of the analysis. Findings were shared in the multidisciplinary OPTIC Study group meetings to facilitate a diversity of perspectives when interpreting the data and identify further avenues for exploration in subsequent interviews.

## Results

Twenty‐one ICU healthcare professionals were interviewed from 20 institutions between November 2023 and April 2024. There were 19 individual interviews and one interview was a nurse and senior doctor pair from the same hospital. Participants included consultants (n = 9); nurses (n = 8); advanced critical care practitioners (n = 2); and allied health professionals (n = 22) (Table 1). Of the 21 participants, 15 were female and ICU experience ranged from 3 years to 30 years: 0–10 years (n = 6); 11–20 years (n = 9); and > 20 years (n = 6). Healthcare practitioners based at different sized hospitals and users and non‐users of current ICU delirium identification tools and care packages were included (Table 1). All interviews were conducted by video‐call (Microsoft Teams; Microsoft Corp, Redmond, WA, USA) and ranged in duration from 45 min to 77 min.

**Table 1:** Summary of hospital characteristics where participants worked.

	Hospitals
	n = 20
**ICU size; beds**	
< 100–499	7
500–1499	8
1500–> 3000	5
**Identification tool use**	
Yes	16
No	4
**Defined care package use**	
Yes	11
No	9
**Geographical location**	
South‐East	3
South‐West	5
London	3
North‐West	3
South Yorkshire	2
West Midlands	1
North‐Yorkshire	1
North‐East	1
Wales	1

The full framework analysis of the tools for identification of ICU delirium is available in online Supporting Information Table S2. Most hospitals in which interviewees worked (18/20) utilised the CAM‐ICU to identify ICU delirium; 1/20 employed the ICDSC; and 1/20 reported not using any tool. Clinicians generally found the CAM‐ICU “*useful*” (Nurse 1), “*easy to do*” (Nurse 5) and felt that “*it works well*” (Consultant 11). However, several participants criticised the tool's questions as “*odd*” (Nurse 1) and noted that “*people feel a bit silly doing it*” (Consultant 10). Many expressed a lack of confidence in CAM‐ICU use, emphasising the need for additional training and a clear care package to support the identification process, highlighted by this statement:

*“…even if I do it [CAM‐ICU] and […] it confirms or refutes my opinion, I'm still left with not a lot of intervention to actually make a difference*.” —Consultant 21

The coded data were grouped into five themes that captured the barriers to providing delirium care: lack of prioritisation; lack of structured ICU delirium care protocols; inability to implement interventions due to physical space constraints and/or lack of resources; loss of experienced staff; and changes in ICU nursing culture. The quotes which support the analysis are in online Supporting Information Table S1.

### Delirium care theme 1: lack of prioritisation

#### Barriers

Competing demands were a key barrier to delirium care (Theoretical Domains Framework: environmental context and resources). Participants from hospitals with extracorporeal membrane oxygenation facilities or neurosurgical units felt delirium was often inevitable and therefore became less of a priority. Hospitals with more elective post‐surgical patients described *“screening negative so often, I think it's [delirium] forgotten in the high‐risk patients”* (Consultant 14). Delirium was more likely to be prioritised if the patient had the hyperactive subtype and posed a risk to staff and their own safety. In contrast, clinicians felt hypoactive delirium was overlooked as patients presented as quiet, drowsy and inactive (Theoretical Domains Framework: beliefs about consequences):

*“… people struggle with that, because they think, ‘Oh, my patient's very compliant and quiet and not saying anything, but actually they're totally terrified inside […] we've introduced […]’ a question to the patient, ‘Do you feel safe?’ to open up that conversation.”* —Nurse 16

#### Facilitators

Some clinicians described ICU delirium as important, with clear delirium protocols. For example, delirium was highlighted as a key safety aspect for the NHS Patient Incidence Safety Response Framework (Theoretical Domains Framework: beliefs about consequences):

*“…this new Patient Safety Incident Response Framework that NHS England […] ((hospital)) is focusing on infection, pressure ulcers, falls and medicine safety and the management of ITU delirium helps to triangulate all of those safety aspects.”* —Consultant 4

Highlighting delirium through hospital posters and discussions in multidisciplinary team, morbidity and mortality meetings and ward rounds increased awareness and staff motivation to prioritise delirium (Theoretical Domains Framework: social influences):

*“It's [delirium] part of the safety brief in the morning.”* —Allied Health Professional 9

Senior staff support was seen as key to helping new nurses prioritise delirium (Theoretical Domains Framework: social influences). The importance of nurses as delirium champions and to advocate for patients was highlighted by Consultant 4:

*“I think the key point of any improvement initiative in a critical care unit is we've got to give the power back to the nurses in leading these things […] they're the ones that really have to push that agenda because they're there advocating for the patients so you know that's going to be the key bit of implementation is making sure the nurses are leading it.”* —Consultant 4

*“…experienced nurses to support and help new nurses identify this is what delirium can look like.”* —Allied Health Professional 7

### Delirium care theme 2: lack of structured ICU delirium care protocols

#### Barriers

Interviewees reported that despite guidelines to screen for delirium once per shift, internal audits suggested that completion was inconsistent:

*“… we know that we are not doing it once a shift, so our big audit showed […] it's just not systematic enough.”* —Consultant 4

Some clinicians described frustration over a lack of a clear delirium management protocol. They felt this devalued screening with the activity perceived as a *“tick box”* exercise (Nurse 5) that would not necessarily lead to a change in clinical management (Theoretical Domains Framework: beliefs about consequences). The current delirium management protocols were described as *“loose”* (Nurse 5), only *“going off people's experience”* (Nurse 2) (Theoretical Domains Framework: behavioural regulation):

*“I think at the moment it's just on recommendations; I don't think there's a particular [delirium] bundle.”* —Allied Health Professional 7

#### Facilitators

Respondents described clear escalation protocols for the pharmacological management of delirium. Some clinicians described moving away from drugs such as benzodiazepines as they felt these made delirium worse (Theoretical Domains Framework: knowledge; beliefs about consequences):

*“I hate benzos for agitation ‘cause I think it just makes it worse down the line’.”* —Consultant 10

Consultant 14 described a *“low threshold for moving off sedatives”* for patients identified as high‐risk of delirium. Despite the existence of pharmacological protocols, nurses reported variability in drug choice among consultants. A poor evidence‐base of the best drugs to manage delirium was often cited as a reason for this inconsistency (Theoretical Domains Framework: knowledge):

*“…every consultant is very different; they all have their set of drugs that they like to use.”* —Nurse 5

Clinicians reported screening was completed more regularly in the presence of digitised prompts and mandated screening that required care plan sign‐off (Theoretical Domains Framework: reinforcement). The use of laminated delirium screening cards provided staff with accessible reminders (Theoretical Domains Framework: memory, attention and decision processes). Some consultants described automated prescribing through order sets to facilitate consistency in the pharmacological management of delirium (Theoretical Domains Framework: memory, attention and decision processes).

### Delirium care theme 3: inability to implement interventions due to physical space constraints and/or lack of resources

#### Barriers

All clinicians expressed a desire to implement non‐pharmacological interventions. However, the hospital or ICU environment and limited staff resources created barriers to implementation. Many clinicians reported that “*noisy*” and “*busy*” ICUs (Consultant 8) made it difficult to protect patients' sleep.

*“…our unit is very noisy. It's too bright. We have sleep packs, but I don't think we use them as much as we should do.”* —Consultant 14

Exposing patients to natural light or the outdoors helped to reorient and regulate their circadian rhythms. However, some units lacked windows, accessible outside spaces or available staff to move patients.

*“I think there's two beds in our entire ITU that have… by a window.”* —Allied Health Professional 7

Lack of access to rehabilitation and psychological support was also noted (Theoretical Domains Framework: environmental context and resources).

*“I really want to have a follow‐up clinic and want to have psychology input […]. The main obstacle is funding.”* —Consultant 8

#### Facilitators

Staff in hospitals with rehabilitative support (e.g. occupational therapists, psychologists and speech therapists) were able to prioritise delirium and non‐pharmacological approaches. A speech therapist noted increased investment in ICU rehabilitation since the COVID‐19 pandemic:

*“It's not always a priority of ours to spend an extra half an hour with the patient […] but they're [delirium team] able to stay with the patient for an hour, talk to them, look at their photos of their dogs.”* —Allied Health Professional 9

### Delirium care theme 4: loss of experienced staff

#### Barriers

The loss of experienced nurses since the COVID‐19 pandemic, high staff turnover and burnout were identified. The departure of experienced nurses led to a loss of skills in recognising signs of delirium, e.g. patients looking *“[like a] rabbit in headlights*” or “*scared*” (Theoretical Domains Framework: knowledge; skills):

*“I don't think they [nurses] are that confident [managing delirium]. I think it comes with experience.”* —Nurse 16

A lack of *“knowledge and understanding”* (Consultant 4) among junior staff and nurses meant that delirium was not a priority on ward rounds (Theoretical Domains Framework: knowledge). Clinicians reported that delirium is only addressed briefly in undergraduate nursing training:

*“I don't know if I'm honest with you how much it [delirium] is taught and on what level because we know that since we started doing it on our […] teaching days, it all seems to be quite new to some of them [nurses].”* —Consultant 4

#### Facilitators

The importance of staff training to equip clinicians with knowledge and skills to diagnose and manage delirium was highlighted (Theoretical Domains Framework: knowledge; skills). One consultant felt training needed to be frequent and targeted, as awareness reduced over time:

*“I think we probably need to do more frequent targeted training because awareness does drop.”* —Consultant 14

Clinicians described a lack of knowledge in the wider team regarding the long‐term impact of delirium and suggested raising awareness would facilitate the prioritisation of delirium care (Theoretical Domains Framework: beliefs about consequences):

*“I think there is a lack of correlation between the person that is in the bed and the person who is going to get better and have to resume their life post ICU stay.”* —Advanced Critical Care Practitioner 13

Clinicians at hospitals with follow‐up clinics observed more post‐discharge psychological and cognitive issues in patients who experienced delirium. Some hospitals provided patient feedback to the ICU to raise staff awareness of long‐term effects, leading to changes such as adjusting curtain colours or avoiding approaching patients from behind:

*“…a lot of patients come back with quite traumatic stories of their delirium […]. As a unit we've tried to implement some changes, like we had blue curtains and a lot of patients thought they were in the sea, so we changed the colour of the curtains.”* —Nurse 5

### Delirium care theme 5: changes in ICU nursing culture

#### Barriers

Some clinicians felt nursing roles were increasingly task‐orientated, reducing time for meaningful patient interaction and reorientation which is key for treatment of delirium (Theoretical Domains Framework: professional role and identity):

*“…they become more technicians rather than nurses. So a lot of the softer skills that nurses would have been doing at the bedside like sitting with your patient and playing a game of cards and trying to engage them, these things don't really happen anymore.”* —Advanced Critical Care Practitioner 13

Frustration regarding sedation practices in patients experiencing delirium has also been expressed (Theoretical Domains Framework: emotions). Over‐sedation (particularly at night) to ensure patient and staff safety was reported (Theoretical Domains Framework: beliefs about consequences):

*“I find it very frustrating if I write in my ward round overnight ‘this patient is not to be sedated’ […] I always find it goes back on.”* —Consultant 12

Nurses reported prioritisation of safety, while some senior doctors felt nurses were anxious that patients who were agitated may self‐extubate. Some nurses perceived there was an expectation to sit with patients with agitation to avoid sedation but reported that providing continuous reassurance during a 12‐h shift was emotionally exhausting and impractical due to other duties (Theoretical Domains Framework: professional role and identity):

*“‘Can't you just hold his hand?’ I said, ‘Well, not really – there's other things that I need to do.’”* —Nurse 2

#### Facilitators

Participants reported nurses lacked confidence to perform sedation holds and required further support (Theoretical Domains Framework: social influences). A consultant described a nurse‐led sedation hold policy that was embedded in routine care:

*“So it's a nurse‐led sedation hold policy that we have in. It's signed off by consultants and then once it's signed off, it becomes nurse‐led and I think those protocols work a lot better.”* —Consultant 14

‘Splitting’ care for a patient with delirium throughout a shift or nursing affected patients in open wards was suggested to support nurses and to stop them feeling isolated:

*“…Should you split the shift and say you get six hours, so you're not constantly exhausted.”* —Advanced Critical Care Practitioner 13

Interviewees highlighted basic nursing skills, including patient communication and attention to causes of agitation (pain, anxiety) as key to delirium treatment (Theoretical Domains Framework: skills). Patient reorientation and reassurance with access to hearing aids and spectacles were considered essential. Reduced agitation with use of communication aids (whiteboards and alphabet charts) was also reported.

*“…you need to have excellent communication skills, because a lot of delirium is caused through patients not understanding what's happening.”* —Nurse 16

Clinicians also highlighted the importance of empathy, recognising how frightening the ICU environment can be to patients. Nurse‐led ‘humanisation’ policies included using a patient's name when requesting information or explaining procedures. Some nurses explained the importance of validating patient experiences to gain their trust, particularly if they had experienced hallucinations.

*“Being empathetic as well – trying to realise that this patient might be a behaving this way because they're scared.”* —Nurse 2

Family members were seen to play an important role in reorienting patients and providing information about usual patient behaviour outside the ICU (Theoretical Domains Framework: social influences). Completing ‘about me’ boards for the patient with family help enabled nurses to better communicate with and reorientate patients:

*“I do think families are really key in supporting with delirium care.”* —Allied Health Professional 7

## Discussion

We found that there is inconsistent implementation of ICU delirium care with systematic barriers that hinder implementation. These barriers exist at all levels, from staff retention to individual preferences of doctors. This is important because delirium is insidious with persistent effects, often long after ICU and hospital discharge. Further work is required to develop solutions that systematically address the barriers and implement the facilitators to improve consistency of care.

The key barriers that influence the use of existing ICU delirium identification tools and care practices include lack of prioritisation; lack of structured care protocols; physical space and resource constraints; staff inexperience; and changes in ICU nursing culture (from non‐technical to technical degree‐level practitioners). Key facilitators include protocols; training and physical prompts; raising awareness; delirium champions; follow‐up clinics; communication; family involvement; support for nurses; and behavioural ‘nudges’ such as electronic prescription order sets.

The themes identified have been shown in previous settings, but not together in a single study and in the UK NHS. A key barrier to delirium prioritisation was the competing demands of ICU. Our study highlights similar barriers reported by ICU staff implementing the ABCDEF bundle [18] in a variety of international settings [10, 11]. Several existing guidelines for ICU delirium provide guidance about what to do, but not how to do it. Whilst this is sufficient for pharmacological components of an intervention, it is insufficient for implementation of non‐pharmacological and multi‐component interventions where staff must perform a specific task. For example, the definition of ‘early mobilisation’ in the ICU and what staff should do to achieve this are not always clear.

A lack of emphasis on delirium within hospital culture contributes to reduced prioritisation in practice [19]. Senior staff are required to promote delirium identification actively; integration into multidisciplinary team meetings and ward rounds; and facilitate the implementation of delirium screening protocols [19, 20, 21]. Loss of experienced staff and associated institutional knowledge is a significant barrier to effective care [22, 23]. This has been particularly stark in the NHS following the COVID‐19 pandemic where 1 in 8 staff left the NHS in the year to 2022 [24]. Although this has now partially abated, staff retention and training remain a significant challenge for NHS ICUs. Communication with patients experiencing delirium is frequently limited and poses a challenge [25, 26]. Validation of patient perception and involvement of family members were identified as strategies that may enhance communication and improve recognition of delirium. However, interventions designed to support communication (particularly for patients who are non‐verbal or whose lungs are mechanically ventilated) remain non‐implemented [27, 28]. There is variability in pharmacological management of delirium [29], even among delirium specialists. This is often influenced by individual clinician preferences rather than adherence to evidence‐based guidelines [30, 31]. Standardised approaches to delirium management have been associated with improved patient outcomes [32], suggesting that a unified, protocol‐driven model of care may benefit both patients and the wider healthcare system.

This qualitative study interviewed a diverse range of ICU healthcare professionals from hospitals of varying sizes and regions across England and Wales, including users and non‐users of delirium identification tools and care packages. This diversity ensured inclusion of a broad spectrum of perspectives and experiences. As such, we suggest that our findings are generalisable across different ICU contexts in the UK. While the number of ICUs sampled was relatively small, they were purposefully selected to reflect key contextual features identified by stakeholders as potentially influencing delirium care. Sample sizes were limited due to the time and resource intensive nature of data collection and analysis. Interviews were conducted by an experienced qualitative health services researcher (RMP) with no previous background in the ICU setting. This enabled a ‘naïve stance’, reducing the risk of bias from pre‐existing assumptions about the ICU setting. A systematic and theory‐informed approach to data analysis, underpinned by the Theoretical Domains Framework, supported a robust categorisation of barriers and facilitators to delirium care implementation.

The study had several limitations. Most participants were female and relatively few allied health professionals were included, potentially limiting the diversity of professional viewpoints. Additionally, participants were drawn from a pool of respondents to a survey on ICU delirium care [13] and may therefore have held a particular interest in delirium, introducing the risk of selection bias. Insights from members of the wider ICU rehabilitation team were limited, and their inclusion may have altered the findings. One interview was paired as respondents wished to be interviewed together; this was not seen to be detrimental to the overall data and during the analysis we explored any differences in their perspectives or experiences. Finally, while the study provides valuable insight into healthcare practitioner perspectives, it does not offer definitive evidence that addressing the identified barriers and facilitators will lead to improved delirium care or reduce the incidence, prevalence or effects of delirium. These can only be truly elucidated by testing in interventional studies, such as randomised controlled trials.

Knowledge of interventions to improve delirium outcomes has been available for many years, and most guidelines remain largely unchanged. For example, there is minimal difference between the 2006 [33] and the 2025 [34] Intensive Care Society guidelines for identification, prevention and management of delirium. To enhance the implementation of delirium care in the ICU, it is essential to combine the barriers and facilitators identified in this study with evidence‐based interventions into a targeted care package, grounded in behavioural science and designed to address these specific challenges. It is difficult to address busy environments and lack of natural light in existing ICUs but this should be considered in future planning of ICU design.

In conclusion, delirium is a priority for ICU staff, but competing demands in a pressured environment, loss of experienced staff and a change in nursing culture from non‐technical to technical tasks are impacting delirium care. Key facilitators include protocol‐driven care; training and awareness; family involvement; and improved communication. Follow‐up clinics and extended rehabilitation may help to mitigate the effects of delirium and provide feedback on longer‐term effects. Addressing these factors directly could aid the design of an evidence‐based intervention for preventing and managing ICU delirium that is both clinically effective and sustainable.

## Supporting information


**Plain Language Summary**.

**Appendix S1.** The OPTIC Study Group.

**Appendix S2.** Topic guide for the interviews.

**Table S1.** ICU delirium Identification Tools Framework Analysis.
**Table S2.** Theoretical domain framework cross‐cutting themes and summary findings.
