# Supplementary material for: Barriers and facilitators to the delivery of delirium care in intensive care units: an analysis informed by the Theoretical Domains Framework
Source: Anaesthesia. 2025 Oct 7;81(2):213–21. doi: 10.1111/anae.70017 (PMC12803597; doi:10.1111/anae.70017)
Supplement: Supplementary file 1 — Plain Language Summary. [file ANAE-81-213-s001.docx]

**Plain Language Summary**

Delirium is when people become very confused or act strangely because of another health problem, such as an operation or an infection. It is common in intensive care units, where very ill people are treated. People who get delirium stay in hospital longer. They may also have problems with thinking and memory after they leave hospital. This study was part of a project to make a new care plan to prevent and treat delirium in intensive care units. Researchers spoke with 21 doctors, nurses and other staff from 20 intensive care units in the UK. They asked about how staff spot delirium, prevent it and care for patients with it. The goal was to understand what helps and what gets in the way.

The study found five problems: some doctors and nurses don’t see delirium as important; many ICU units don’t have clear plans to how to deal with delirium; many units don’t have enough staff; experienced staff are leaving; and nurses are now doing more technical tasks instead of traditional caring roles. But the study also found things that can help. For example, having a clear care plan for delirium, training staff often and making them aware of delirium, or having staff who just focus on delirium can help. Also, good communication, setting reminders for staff to check on patients, involving families in care and checking on patients after they go home. In short, most doctors and nurses know that delirium matters but they struggle to deal with it properly. To make things better, intensive care units need clear care plans, more training and more family involvement.
