# Supplementary material for: Barriers and facilitators to the delivery of delirium care in intensive care units: an analysis informed by the Theoretical Domains Framework
Source: Anaesthesia. 2025 Oct 7;81(2):213–21. doi: 10.1111/anae.70017 (PMC12803597; doi:10.1111/anae.70017)
Supplement: Supplementary file 2 — Appendix S1. The OPTIC Study Group. [file ANAE-81-213-s004.docx]

**The OPTIC Study Group**

Mike Grocott^1^, Emma Hopkins^2^, Alicia O’Cathain^3^, Paul Moran^4^, Claire Black^5^, Cathrine McKenzie^6^, Andy Gibson^7^, Susie Robinson-Molloy^8^, Burak Kundakci^9^, Andrew Booth^3^, Katherine L Jones^9^. Sarah Smith^10^, James Long^11^, Molly Potter^11^, A Francis Johnson^12^, Susie Robinson-Molloy^8^,

1. Professor, University of Southampton, Southampton. UK
2. Research Sister, University Hospitals Bristol and Weston NHS Foundation Trust, Bristol. UK
3. Professor, School of Health and Associated Research, University of Sheffield, Sheffield. UK
4. Professor, Bristol Medical School, University of Bristol, Bristol. UK.
5. Physiotherapist, University College London NHS Trust. London. UK
6. Associate Professor, University of Southampton, Southampton. UK
7. Associate Professor, University of the West of England. Bristol. UK
8. Research Associate, Bristol Medical School, University of Bristol, Bristol. UK.
9. Research Associate, School of Health and Associated Research, University of Sheffield, Sheffield. UK
10. Associate Professor, Department of Health Services Research & Policy, London School of Hygiene & Tropical Medicine, London. UK
11. Patient Contributor, OPTIC Study Group, University of Bristol, Bristol. UK
12. Trials Co-ordinator, Intensive Care National Audit and Research Centre, London. UK
