# Supplementary material for: Barriers and facilitators to the delivery of delirium care in intensive care units: an analysis informed by the Theoretical Domains Framework
Source: Anaesthesia. 2025 Oct 7;81(2):213–21. doi: 10.1111/anae.70017 (PMC12803597; doi:10.1111/anae.70017)
Supplement: Supplementary file 3 — Appendix S2. Topic guide for the interviews. [file ANAE-81-213-s003.docx]

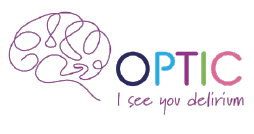
**

**

**Appendix S2. Topic Guide for the interviews**

**OPTimising the prevention, identification and management of ICU Delirium (OPTIC)**

## Topic GuidE: interviews with ICU healthcare professionals BASED ON the Theoretical Domains Framework

**Screening**

1. Can you tell me about how delirium is detected and managed in your ICU?
2. Whose responsibility is it to carry out screening and management of delirium in your centre? *Does this work well, do you feel it should be done differently?*

*(TDF: Social/ professional role and identity; Social influences)*

1. How confident do you feel you/your colleagues are at diagnosing and managing delirium? *(enough training/ skills/ emotions or feelings about it?)*

*(TDF: Beliefs about capabilities)*

1. How do you currently screen for delirium in ICU?

*(Categorise people into hyperactive / hypoactive / mixed any implications for treatment?)*

1. What do you think of the current screening instruments? Can you tell me about any other screening tools you have tried?

*(Easy/hard to use? Why/ when do you use it?*

*Are you confident using them?*

*Are you confident that it picks up delirium?*

*What are the good aspects of the tool? What could be improved/ is missing from the tool?*

*If no tool: why?) (TDF: Knowledge)*

1. Do you believe the delirium screening tools are valuable? Why?

*(TDF: Optimism; Beliefs about consequences)*

1. Do you feel there is a need for a new screening tool and if so, what features do you think it should have? *(quicker, more sensitive to different delirium subtypes, etc)*
2. Is it easy or difficult to perform delirium screening and why? *(E.g. agitated pt, specific tool?, screening instruments available, staffing levels, lack of training, habit; ward environment?)*

*(TDF: Environment, context, and resources; Behavioural regulation)*

1. Do you have any specific protocols in place for screening (do you think they work as well as they could?)

*(TDF: Environment, context, and resources; Behavioural regulation)*

1. What if anything stops you from routinely screening? *E.g. Forget, staffing levels, grade; competing priorities; sufficient resources; knowledge/lack of training; concerns/worries; tools don’t work/ difficult to use?*

*(TDF: Memory, attention, and decision processes; Emotion; Behavioural regulation; Reinforcement)*

1. What if anything encourages you to screen routinely? What from your perspective would help? (If we incentivised people (CQUINS) do you think that would that help?)

*(TDF: Memory, attention, and decision processes; Emotion; Behavioural regulation; Reinforcement)*

1. How easy or hard is it to get to the route cause of what may be contributing to delirium e.g. pain, constipation, pressure ulcers?
2. What do you think are the necessary skills to identify delirium in ICU patients? *E.g. Clinical, communication, decision making skills of doctors and patients? Do you believe you/ your colleagues have all the necessary skills you need?*

*(TDF: Skills)*

1. Is delirium discussed in multi-disciplinary meetings what part if any does teamwork play in management? *(Conflicts regarding management?)*

*(TDF: Social/ professional role and identity; Social influences)*

1. In your experience, is it important to detect and treat patients who may hide or feel they can’t discuss their delirium?”

*Should delirium be detected retrospectively?*

1. Is delirium managed differently for different types of patients e.g. age groups and cultures? If so how?
2. Do you ever seek the opinions of colleagues/ patients’ family members when assessing delirium?

*Prompts: Family members ever informed about the possibility of delirium?*

*(TDF: Social/ professional role and identity; Social influences)*

**Prevention and Management**

1. Can you tell me about any specific care packages/protocols that you have in place in your unit for the prevention or management of ICU delirium? (Do they work as well as they could?)

*(TDF: Knowledge, Environment, context, and resources; Behavioural regulation; Goals; Intentions)*

**If no care package:**

1. Why not? Are you planning to implement one? If not, why not *(resources, lack of evidence etc…)*

**If they have existing care package:**

1. What if any aspects of your delirium care packages/ protocols work well? *(Easy to implement/ show clear improvement)*
2. What aspects of delirium prevention and management do you find particularly challenging? Please give some examples. *E.g. do you ever forget? Lack of resources; knowledge; concerns/worries*

*(TDF: Memory and decision processes; Environment; Behavioural regulation; Reinforcement; Emotion)*

*(TDF: Beliefs about capabilities)*

1. What, if anything might help with overcoming these challenges? *(e.g. training, different tools, more time & resources)*

*(TDF: Beliefs about capabilities)*

1. What would encourage you to implement delirium care packages/protocols routinely?

*(TDF: Memory, attention, and decision processes; Emotion; Behavioural regulation; Reinforcement)*

*(TDF: Skills)*

1. What influences your decision to use pharmacological interventions to manage delirium *(e.g. antipsychotics, benzodiazepines, sedatives) Which do you use and why? Would you be willing to reduce use/change your use of pharmacological interventions? Goals around use of pharmacological interventions?*

*(TDF: Decision processes; Behavioural regulation; Reinforcement; Emotion)*

1. Do you implement non-pharmacological interventions? Which ones?

*(e.g early mobilisation, removal of catheters, sleep hygiene, re-orientation, family visiting)*

1. What makes it easier or harder for you to implement non- pharmacological interventions?

*(TDF: Skills;* *Environment, context, and resources; Behavioural regulation, Memory)*

**Overall**

1. Do you have regular staff training for screening or managing delirium?

*(Is there any specific training in your department? Mandated or more ad-hoc?)*

1. Is there anything you worry about/ makes you nervous or frustrated when managing delirium in patients?

*(TDF: Emotions)*

1. In what ways do you think diagnosing and managing delirium in patients might affect their long-term outcomes?
2. How much of a priority is the prevention and treatment of delirium a priority on your unit?

*(TDF: Optimism; Beliefs about consequences)*

1. What are the advantages or disadvantages of detecting and managing delirium?

*Improve outcomes for patients? Is the impact of delirium understood by colleagues?*

*(TDF: Optimism; Beliefs about consequences)*

1. Have you changed the way you care for patients with delirium in ICU over the past few years? And If so, how and why?

*(TDF: Action planning)*

1. Is there anything else you would like to tell me about barriers and facilitators to the management of ICU delirium? Anything you feel we have missed?*(TDF: Optimism; Beliefs about consequences)*
