# Supplementary material for: Barriers and facilitators to the delivery of delirium care in intensive care units: an analysis informed by the Theoretical Domains Framework
Source: Anaesthesia. 2025 Oct 7;81(2):213–21. doi: 10.1111/anae.70017 (PMC12803597; doi:10.1111/anae.70017)
Supplement: Supplementary file 4 — Table S1. ICU delirium Identification Tools Framework Analysis. Table S2. Theoretical domain framework cross‐cutting themes and summary findings. [file ANAE-81-213-s002.docx]

**Table S1.** Theoretical Domains Framework domains, cross cutting themes and summary findings

| **TDF Domain & Themes with Summary Findings** | **Sample Quotes** |
| --- | --- |
| 1. **KNOWLEDGE**   **Loss of experienced staff**   - Interviewees themselves demonstrated good knowledge of delirium, types and impact. However, it was felt that there had been a loss of experienced staff and more junior staff lacked knowledge about delirium, its importance and particularly its long-term impact on patients.   **Training**   - Training to increase the knowledge of delirium was variable between units.   **Family involvement to help reorientate patients**   - Family is an important source of knowledge about patients and their ‘normal behaviour’.   **Delirium not prioritised in the ICU**   - There was felt to be a lack of an evidence base for the best drugs to use to treat delirium which often led to a lack of consistency in pharmacological management.   **Structured delirium care**   - Some units had clear care pathways and protocols for identifying and treating delirium. Others described a lack of protocols or action plans for when someone is diagnosed with delirium. | NUR3: “So it can present very differently. So you can have hypo Delirium and hyper Delirium. A hypo Delirium is a lot harder to identify”  CON4: “…our big audit showed that we weren’t doing it [CAM-ICU] and the reasons – well we do sometimes, it’s just not systematic enough and it’s picked up by the nurses but then the nurses probably don’t understand how important it is”  NUR2: “There’s no real sessions.’ There is a delirium, but it’s not particular to ICU – there is a delirium package that we can learn, but it’s not mandatory.”  NUR18: “Yes, so we do regularly try and do teaching about delirium”  CON15: “Sometimes it's useful to talk to families because you might find out that the patient doesn't like certain types of music, or has fears of being cold, or being hot, all sorts of range of things you find out about patients. And actually that may be making their experience for them difficult, and almost contributing to their delirium, or making it worse, or more prolonged.”  CON15: “I think it's because there is no definite good or proven best drug to use for management of delirium, and I think that's contentious at times”  AHP7: “I think at the moment it’s just on recommendations, I don’t think there’s a particular [delirium] bundle or protocol at the moment.”  CON11: “protocol is first you do your CAM ICU assessment. If it's positive you then address all the non-pharmacological measures and only if the patient is a risk to themselves or to others do they start down a pharmacological management” |
| 1. **SKILLS**   **Loss of experienced staff and skills to detect and treat delirium**   - There was felt to be a loss of experienced staff and relevant skills for diagnosing and treating delirium since COVID-19 pandemic. - The ability to spot observable signs of delirum e.g “wild eyes”, rabbit in headlights” as well as patients not following the expected trajectory were important skills that were felt to come with experience, particularly hypoactive delirium which is more difficult to detect. - Interviewees themselves felt confident using the CAM-ICU. However, they felt that many nurses lacked competence and confidence in using the CAM-ICU.   **Changes in ICU nursing culture**   - There was felt to be a shift in nursing culture with nurses becoming more assessment orientated and placing less importance on the basic interpersonal skills of sitting with, talking to patients and reorienting them.   **Communication is key to reorienting patients**   - Communication skills were felt to be key to reassure and reorientate patients to help manage delirium. - Being calm and empathetic and understanding how disconcerting ICU can be for patients was important to validate patient experiences, build trust and increase patient willingness to disclose any worries. | CON15: “We have gone through a phase where we've employed quite a lot of new staff following COVID. And yes, it's taken some time to train those staff up, and I think yes there is a difference. If we have a particular patient, difficult with agitation and delirium, we’ll tend to put a more experienced nurse with that patient.”  NUR17: “We see a lot of people with delirium but I don’t think we necessarily are prepared with the skills to deal with that.”  NUR16: “But then there's also the physical signs that you would look for around inattentiveness, the wild eyes that we always see happen in the paranoia.”  NUR3: “Then picking up delirium comes really because they’re not following the trajectory you’d expect and to know what to expect comes with experience doesn’t it.”  NUR19: “I think certain people are, so I feel very confident, but it’s been a passion of mine for a few years”  NUR16: “I don't think they [nurses] are that confident. I think it comes with experience, and I think it depends on experience”  CON11: “If the nurses aren't trained and confident with the CAM ICU tools then you run into some difficulties and we may get more patients here kind of classed as unable to be assessed”  NUR16: “I think it is a generational shift, and it's not just in nursing, it’s in medicine, it's in lots of professions where people are maybe not coming into the profession for what they thought, and people don't see the value necessarily of just talking and touching a patient, and learning that observation skill of walking into a room and thinking, ‘Oh, you're not right.’”  NUR5: “I think it’s just about giving as much reassurance […] it’s about trying to stay calm and actually revalidating what they’re seeing is real to them at that time and not fighting with it.”  NUR6: “I think you have to have good communication skills with the patient […] if they’re looking in the corner all the time and they’re looking very scared. It’s like, “Can you see anything over there, are you worried about anything?”  NUR17: “I find the problems with it really are the fact that there can be communication issues, the patient might not hear you, they might be in such a state of anxiety and fear that they don’t really understand what you’re saying” |
| 1. **SOCIAL PROFESSIONAL ROLE AND IDENTITY**   **Delirium champions who prioritise delirium care**   - Interviewees themselves appeared to be delirium champions and taking a personal interest in delirium implementing projects to improve delirium care in their hospitals.   **Changes in ICU nursing culture**   - There was a sense of a loss of the ‘traditional’ nursing role: sitting at the patient’s bedside, talking to them to reorientate them and manage delirium. Nurses were felt to be more assessment and data driven. | *NUR3: “So I’m particularly interested in Delirium because I do the follow-up clinic”*  *CON4: “…now that I’m a Consultant and I’ve been reading all about the Society of Critical Care Medicine’s Liberation bundle and all their delirium and sedation work”*  NUR13: “So then they become more technicians rather than nurses. So a lot of the softer skills that nurses would have been doing at the bedside like sitting with your patient and playing a game of cards and trying to engage them, these things don’t really happen anymore. They are more consumed with collection of data.”  CON12: “I think cultural identity of what nurses think they do and what their role is has changed quite significantly.” |
| 1. **BELIEFS ABOUT CAPABILITIES**   **Loss of experienced staff**   - Interviewees themselves felt confident in diagnosing and treating delirium. However, due to the more junior workforce it was felt more generally that there was a lack of confidence particularly in: using the screening tools e.g. CAM-ICU, dealing with agitated hyperactive delirious patients and carrying out sedation holds. | NUR17: “I feel quite confident in diagnosing it, using the flow chart. Actually managing it, if I’m being really honest, I don’t feel completely confident in managing it, because it’s the unknown really.”  NUR19: “I think certain people are, so I feel very confident, but it’s been a passion of mine for a few years”  NUR6: “I don’t know whether people necessarily are confident in using it [CAM-ICU] even though there has been teaching on the unit.”  NUR19: “I would say a good 80% are probably not confident [in diagnosing delirium], […] of everything they have to learn it’s [delirum] probably way down the list of things that they have to learn. That will take time to build up.” |
| 1. **BELIEFS ABOUT CONSEQUENCES**   **Delirium champions who prioritise care**   - Interviewees were able to talk about the long-term impact of delirium and the importance of identifying and treating it to minimise long term consequences.   **Loss of experienced staff**   - A feeling that for more junior staff there is a disconnect between the patient in the bed and the patient that leaves ICU and has lasting consequences of delirium. - Bigger hospitals with more complicated ECMO and emergency patients described a feeling that delirium was inevitable and therefore not preventable and therefore screening was felt to be less valuable.   **Lack of clear delirium protocols**   - In hospitals that lacked clear delirium protocols, screening was less valued as identification didn’t necessarily lead to treatment.   **Delirium not prioritised on the ICU**   - Participants described the impact particularly of hyperactive delirium on patient and staff safety and therefore there was aften a tendency to sedate patients in order to provide this safety. - It was felt that often hypoactive patients are easier to care for and therefore this type of delirium is missed. | NUR1: “((Name)) has done a lot of work on delirium because she runs the follow-up clinics – so she’s got a big interest in the long-term effects of delirium. So, we do the training between us for delirium”  CON4: “I think across the board intensive care teams have much more junior and inexperienced nurses compared to historically”  NUR13: “It’s down to experience. You have to understand the impact that it’s going to have on that person’s life when they leave hospital. You are not providing care just for that moment in time. You are setting it up for the next 18 months…”  *CON10: “maybe 40% of our patients are neurosurgical. It adds to people’s, um, not jaded nature but people who, ‘oh well, they’re going to get delirium whatever because they’ve bashed their brain.’”*  NUR13: “I think everything could be done better. I think it is a tick box exercise. It doesn’t mean anything meaningful”  CON12: “There’s definitely a tendency to keep patients deeper than they need to be. There’s this great fear of accidental extubation”  CON8: “…people are less bothered by people who are sat in the bed not bothering.”  NUR16: “The people who sit quietly have the long-term effects, because nobody picks it up.” |
| 1. **REINFORCEMENT**   **Follow up clinics to increase staff understanding of the long-term impact of delirium.**  In hospitals that had follow clinics, they saw the consequences of delirium on patients and the long-term impact was more clearly understood. | CON14: “if you’re delirious, and we’ve certainly seen that in the follow-up clinic clearly where they were struggling with even hypermotor delirium. There are more psychological morbidity and cognitive issues afterwards.”  CON15: “…they tend to either construct an email around what's happened [from follow up clinics], and send that round to the staff” |
| 1. **INTENTIONS AND GOALS**   **Changes in ICU nursing culture**   - Most interviewees felt it was important to move towards more non-pharmacological interventions and reduce pharmacological interventions. However, in practice it was often difficult to manage agitated patients and this often let to sedation and drug creep with more and more drugs being tried. | NUR3: “Yeah we do try and avoid them [drugs]. So we realise that adding more chemicals to the brain is not going to help necessarily. But we’re victims of these patients… if we could get them outside every day and get relatives there for the bulk of the shift, we might have more success with non-pharmacological interventions. But those things aren’t always possible or completely effective.”  CON14: “We have 2021 guidance and it focuses quite heavily on prevention. […]If we are predicting that the patient’s going to have some issues in the early part of wean phase, we’ve got a low threshold for moving off sedatives and moving onto dexmedetomidine quite early”  NUR6: “…it’s more difficult implementing the non-pharmacological ones, especially if they’re very agitated and very confused. Trying to talk somebody down takes a lot more time than actually just giving them a dose of whatever to take the edge off things.” |
| 1. **MEMORY, ATTENTION AND DECISION PROCESSES**   **Delirium not prioritised on the ICU**   - There was variation in whether delirium assessment, prevention and management was embedded in practice. The critical nature of ICU meant there were often more important competing priorities and delirium was sometimes forgotten. - Delirium was more likely to come to the attention of staff when patients became agitated and this had implications for safety.   **Digital / physical prompts to facilitate regular screening**   - Hospitals using physical prompts such as check boxes on charts or laminated CAM-ICU cards helped prompt delirium screening. - Computerised protocols with mandatory care plan sign off facilitated screening. - Prescribing through order sets facilitated consistency in the drugs used to treat delirium. | NUR6: “we document an awful lot, every single hour and so if they’ve not scrolled down to the bottom of the page then they might well miss it [delirium]”.  CON15: “…we've got delirium protocol online, I don't know if anyone really looks at it or uses it.”  NUR2: “…it’s only when someone starts becoming a danger to themselves – or they’re gonna end up pulling out lines – or anything – that there is any screening done.”  NUR5: “…we’ve got a lot of new starters so we’ve given them all little cards which has got the CAM-ICU and the sedation scoring on it”  CON8: “We’ve got a guideline for pharmacological and nonpharmacological management of delirium”  NUR19: “With our new electronic patient record system there is no way of flagging it up at the moment, it’s something that we’re working on and so it is the bedside nurse’s responsibility yet I think unfortunately because there’s no kind of reminder it’s not high up on the list.”  NUR18: “I think having this digital solution that kind of forces people to do it every 12 hours has made it a lot easier with compliance to do it.”  CON8: “We try and push everybody towards prescribing through our order sets rather than through their own experience.” |
| 1. **ENVIRONMENTAL CONTEXT AND RESOURCES**   **Inability to implement interventions due to physical space constraints and lack of resources**   - The critical nature of ICU makes it difficult to prioritise delirium care due to more important competing demands. - The ICU environment makes it challenging to implement particularly non-pharmacological management. Noise and admissions make it difficult to protect sleep, lack of natural light and lack of staff to take patients outside make it difficult to regulate circadian rhythms. - Lack of access to wider rehabilitation team members make it difficult to implement non-pharmacological approaches such as sitting and talking to patients to orientate them, taking patients outside, OTs to sit patients up and encourage eating, psychologists to explore patient hallucinations or worries. - If delirious patients are not classed as level 3 this can make it extremely difficult to care for an agitated patient. - Side rooms provide a quite environment for a patient to promote sleep however this can leave nurses isolated when managing a hyperactive delirious patient. | CON10: “I think it’s [delirums’] a major issue that a lot of people just kind of think well it’s not sepsis so it’s not going to kill you immediately so why do we worry about it?”  NUR5: “…we’re very bad in intensive care, we’re very noisy, we like a lot of lights on and it’s just about making it a bit more human.”  NUR18: “’Im like, “Please don’t move them, please don’t move them to the place where there is no daylight.[…]. That’s more distressing. I find the whole thing distressing really because I see that the environment of our department does cause delirium or make it worse.”  NUR18: “I think like staffing levels, staffing experience, so for example taking someone outside takes a number of staff if someone’s still ventilated”  CON10: “We don’t have any occupational therapists on our unit”  AHP7: “we really need psychology to come in and I think once we get that approved, we’re hoping to have I think monthly follow-up clinics for patients.”  NUR17”…level 2 patients can be looked after by one nurse. So again, that’s very difficult then to initiative that delirium care for them.”  NUR16: “units are getting larger and larger, lots of cubicles, staff feel isolated. […] very difficult to manage them when they’re delirious. It all sounds great having individual rooms; it leaves staff feeling really vulnerable.” |
| 1. **SOCIAL INFLUENCES**   **Clinical staff prioritising delirium**   - There were clear interprofessional influences on the prioritisation of delirium. Where senior staff prioritised delirium and regularly enquired about it or it was regularly discussed on ward rounds and MDT meetings this promoted the identification and management of delirium.   **Delirium champions who prioritise delirium care**   - Delirium champions optimised practice.   **Family involvement to help reorientate patients**   - Families were identified as important to identify, prevent and manage delirium in patients.   **Supporting nurses**   - Nurses require senior support with sedation holds | CON14: “We have a nurse who leads on delirium, and she does push it and there’s guidelines in place.”  NUR19: “We have certain consultants who will bring it as part of their ward round […] “Is this patient delirious?”  *NUR17: “…we don’t actually discuss it [delirium] in an MDT.”*  NUR18: “…like an MDT huddle and if someone was delirious it would be highlighted and we would discuss what to do”  CON4: “…now that I’m a Consultant and I’ve been reading all about the Society of Critical Care Medicine’s Liberation bundle and all their delirium and sedation work”  CON8: “We have a fulltime nurse educator so that’s [delirium] one of the things that she’s been championing”  NUR6: “I think you need to get to know the family so that you can get to know the patient better if they’re not able to tell you what their needs are. So you can do those things such as hearing aids, glasses, walking aids, teeth, all that is essential for communication which will help relieve delirium if you can communicate and they can communicate back.”  CON10: “getting the family to help calm people down when they’re agitated and hyperactive is really important.”  CON11: “…understanding what those barriers might be for that nurse and trying to work out how to address them and help them […] Sometimes it's about reducing the sedation when you're there and you're present so that you've seen what the patient is like underneath.” |
| 1. **EMOTIONS**   **Changes in ICU nursing culture**   - Some clinicians expressed frustration that delirium protocols were not followed or that patients were over-sedated, particularly at night. - Interviewees talked about fear and anxiety in nursing staff about dealing with hyperactive delirious patients accidental self-extubation of patients. - Disagreement between different clinicians in the pharmacological management of patients. Use of drugs was based on clinician preference and lack of clear guidelines for drugs was a key barrier. | NUR13: “I find it quite frustrating that we don’t all have a structured approach to delirium.”  CON12: “There’s definitely a tendency to keep patients deeper than they need to be. There’s this great fear of accidental extubation”  NUR16: “we are trying to do the restraint reduction where we don't chemically restrain as much, that's challenging. Often the day team will reduce the medication, and then it leaves the night team in a position of battling it out with the patient,”  CON15: “I think it's because there is no definite good or proven best drug to use for management of delirium, and I think that's contentious at times is that some physicians feel that patients shouldn't be on any medications, and there can be conflict from the nursing staff because it can be difficult to manage patients who are very delirious and hyperactive.”  NUR18: “Yeah it’s just different doctors having different ideas about what medication to use. So then someone might start something and then someone else will come in and change it, stop it, so there’s a bit of inconsistency.”  CON15: “if there was a protocol that was a very well-evidenced based protocol, that would be good to teach nurses, but I think sometimes they find it hard to know what to do, because there's so much variation in terms of what drugs are used.” |

**Table S2.** ICU Delirium Screening Tools Framework Analysis

| **Participant I.D** | **Screening tool used and frequency**  **Planned and actual use** | **Good aspects of current screening tools** | **Problems with current screening tools** | **Is a new screening tool needed?** | **Ideal features of new tool** |
| --- | --- | --- | --- | --- | --- |
| **NUR1**  **CAM-ICU** | **CAM-ICU every shift**  *NUR1: “So, every patient – every day – is screened using the CAM-ICU tool. So that’s done every shift”* | **Useful**  *NUR1: “I think it’s a useful tool […] I think it helps us to identify people that are suffering.”* | **Questions are odd**  *NUR1: “I think some of the questions are a bit odd – that people wouldn’t necessarily understand anyway”*  *NUR1: “ …so, to me, ‘Can you pound a nail with hammer?’ That is not how I would speak.”*  **Not sensitive**  *NUR1: “…people can pass the CAM-ICU and you still know that they are confused.”*  *NUR1: “I go to the unit handover every day and they’ll say, ‘The patient is ‘Pass’ on the CAM-ICU, but doesn’t know where they are”.*  **Makes patients feel stressed**  *NUR1: “… ‘Squeeze my hand when you hear the letter A’ – but people feel stressed about these things, I think. If you’re dyslexic – or not terribly well – you’re not particularly literate – I think those are difficult things to do.”* | **Unsure**  *NUR1: “I suppose it could be more sensitive – I’m not sure how. If you wanted me to re-design a CAM-ICU, I would really have to sit and give it some thought!”* | ***General Qs on year***  *NUR1: “…you could perhaps ask – or what year it is! ‘Cause, if someone’s thinking it’s 1962, then – obviously – they’re a bit confused”* |
| **NUR2**  **No screening tool used.**  **RASS sometimes** | **None_RASS sometimes**  *NUR2: “We don’t officially have any tools […] we do rudimentary use RASS”*  **Screening only when pt very agitated**  *NUR2: “…it’s only when someone starts becoming a danger to themselves – or they’re gonna end up pulling out lines – or anything – that there is any screening done.”* | **Screening gets doctors into action**  *NUR2: “It does get the doctors into action. […] when you do get hold of them, and you say, ‘Oh, his RASS is ‘Plus-3,’ and we’re gonna lose lines if you don’t come and review this patient.’ ‘Oh, right – okay.’ For some doctors, it seems to be… if you give them that clinical justification – rather than just, ‘Oh, well – can you just sit with them, and just hold their hand?’ It’s like, ‘No – no.’ It’s scaring me!”* | **RASS is subjective:**  *NUR2: “…what somebody might class as ‘Threatening behaviour,’ another person might say, ‘Oh, they’re fine – they just need to calm down a bit.’ So, it’s very subjective, in my opinion – that’s the one thing where it does fall down.”* |  | **Measure change from baseline, objective**  *NUR2: “It would be good to have perhaps a baseline, so it can monitor the change – people’s behaviour a little better than what RASS does. It would also be a bit more objective, rather than subjective – but then we’re talking about behaviour – where it is a very subjective subject – I suppose! – people’s behaviour. What is acceptable for person is totally unacceptable for other people.”* |
| **ACCP3**  **CAM-ICU** | **CAM-ICU when delirium suspected**  *NUR3: “…if we started to consider that there is Delirium we would do a CAM ICU test.”* | **Quick and effective**  *NUR3: “CAM ICU is really quick and effective to do, so yes I like it.”*  **Picks up delirium**  *NUR3: “…they have to be not delirious enough that they can actually engage in the process. But if they’re not able to engage in the process then you’re almost certain it’s Delirium.”* | **Doesn’t pick up hypoactive delirium**  *NUR3: “The CAM ICU doesn’t really work for hypo acted Delirium, what tool would I mean. So I don’t see that as a failure of the tool.”* | **No-CAMICU ideal for high functioning pts in ICU**  *NUR3: “… you’ve got to be able to have quite a high level of executive function for example to be able to answer the questions. But you’re only really in Intensive Care if you’re functioning at that level already. I talk about dementia but we have so few patients with dementia in critical care because they tend to not be for full escalation. […] So for our population, the CAM ICU works really well. At ward level, it’s probably entirely different. But for Intensive Care it’s ideal.”* | **Orientation important**  *NUR3: “…a delirious person can follow commands but they probably wouldn’t reorientate to a place and time.”*  **Ability to identify family**  *NUR3: “…they show worrying signs which is, they might follow commands but they’re still a bit muddled, or they’re not identifying family members”*  **Unpick erratic fear driven behaviour from delirium.**  *NUR3: “People who are traumatised, which obviously everyone is by the time they end in intensive care, aren’t being themselves, aren’t thinking coherently and can have quite erratic behaviour which is fear driven rather than Delirium driven”*  **Explore and treat other route causes of agitation- pain, anxiety, personality**  *NUR3: “…working with the therapy team, we sort of unpicked that she’s not delirious, actually what she is, is a combination of institutionalised… quite an erratic personality anyway and very in pain and very anxious. Then so what had been labelled as delirious by the medical team again and again, in night and day handovers, is actually not Delirium, it’s a combination of other emotional things. Which is really important because they will not respond to pharmacological management”* |
| **CON4**  **CAM-ICU** | **CAM-ICU per shift but audit showed not completed**  *CON4: “…our big audit showed that we weren’t doing it [CAM-ICU] and the reasons – well we do sometimes, it’s just not systematic enough and it’s picked up by the nurses but then the nurses probably don’t understand how important it is”* | **Its fine**  *CON4: “CAM-ICU is probably fine when patients are as I said awake, it’s just when they’re sedated it’s a bit tricky”* |  | **Unsure**  *CON4: “I don’t know from a research perspective what’s out there to detect delirium from a monitoring perspective other than a clinical screening tool. I don’t really know how you would do it”* | **Entropy or Bis monitoring**  *CON4: “I use Entropy or Bis which measures people’s brainwaves and you can see how deeply sedated people are so they can – and during COVID when we realised how delirious a lot of these patients were, I did ask if we could bring in some Bis monitoring and just see what was happening with the brainwaves”* |
| **NUR5**  **CAM-ICU** | **CAM-ICU every shift**  NUR5: “*So we use* *CAM-ICU on our unit. Every patient gets – they do that every day, well every 12 hours we do that unless there’s any changes or causes for concern where we think there’s a change in their understanding.”* | **OK-it works**  *NUR5: “I think for the CAM-ICU it’s okay, it works.”*  **Easy to use, used it for a long time**  *NUR5: “…it [CAM_ICU] is quick and easy to do […] I think we’ve just used it for so long that’s just what we use.”* | **Nurses need more training on it- some qs can be misunderstood**  *NUR5: “…it [CAM-ICU] asks whether or not you’re able to actually do a proper assessment on the patient. I find that if, sometimes when they are confused and agitated people will tend to put down, “Unable to do as patient confused.” So it’s getting their understanding, actually that means they’ve probably got a bit of delirium and just a bit more education that we’re trying to do.”*  ***Pts feel qs are odd and need educating***  *NUR5: “…patients don’t like it, they quite often turn around and say to me, “Why do they ask me such stupid questions”*  **Not always sensitive**  *NUR5: “I think sometimes it does miss it, I think sometimes just by having a conversation with them you can probably pick it up”* | **No- better training on tools needed**  *NUR5: “…it’s just getting them educated on how to do it properly I guess.”* | **Not clear- pt once suggested a range of questions**  *NUR5: “No I don’t think so, the only other thing that a patient has mentioned is, “They ask me the same thing every time, surely there’s other questions.””*  **Need to talk to pt_pt restless_rabbit in headlights**  *NUR5: “Sometimes it’s the lack of eye contact they give you, or if they’re just not able to focus on what you’re saying, if they’re kind of looking around the room quite a bit sometimes. We always call it a rabbit in the headlights, they have that expression and you look at them and you think there’s something not quite right there. When they’re just generally very restless in the bed or trying to get out of bed or asking the same questions over and over again.”* |
| **NUR6**  **CAM ICU**  **RASS** | **CAM-ICU but not regularly completed**  *NUR6: “We are supposed to do CAM-ICU once a shift and RASS every four hours for a sedated, ventilated patient, however I know that’s not done because having done a research study on it recently I know it’s not documented frequently enough.”* |  | **Odd questions**  *NUR6: “That’s the one that the unit has adopted. I don’t think it’s particularly great, like you say, “Fish in the sea, does a stone float?” I’m not sure, I think if you’re a little bit muddled with painkillers and being ill and then somebody starts asking you that, even I would go, “What are you on about?” So I don’t know whether it’s really assessing adequately enough essentially, I don’t know what would.”*  **Lack of confidence using it**  *NUR6: “I don’t know whether people necessarily are confident in using it even though there has been teaching on the unit.”* | **Potentially, not sure what**  *NUR6: “I think there could be something better out there but I’m not quite sure what.”*  *NUR6: “Yes, if you could come up with something better which is obviously what you’re trying to do, then yes go for it. What features? It needs to be quick and easy, it needs to be easy to remember, it needs to be easy to document.”* | **Observable signs of patient looking scared**  *NUR6: “…if they’re looking in the corner all the time and they’re looking very scared. It’s like, “Can you see anything over there, are you worried about anything?””*  **Family assessment should be included**  *NUR6: “I think it’s very important to include the families in those sorts of assessments because they’ll know, “She’s talking complete gibberish, she wouldn’t usually be saying this.” You’re like, “I think she’s been fine all day.” It’s only close family members or loved ones that would actually detect and pick up those subtle changes really.”* |
| **AHP7**  **CAM-ICU** | **CAM-ICU not routinely completed**  *AHP7: To be honest it’s probably something that needs to be detected more […] there’s also the CAM-ICU prompt for both the doctors during their daily ward round and also the nursing, but the nurses have a separate set of documentation that they document on whether it’s positive or negative for CAM-ICU, but it’s not often filled in”*  *AHP7: “we basically look through all the notes and see where the delirium’s documented for that day and if it’s documented in the doctors’ ward round and the nursing handover and I think we’ve done it four or five times and we haven’t seen anyone document any across all our 30 beds.”* | **OK**  *AHP7: “I think it’s okay* | **Not well translated into other languages**  *AHP7: “I think from that it does have its challenges and sometimes we say oh yeah, they’re CAM-ICU positive but actually it’s because with some of our patients where English isn’t a first language and it’s translated into for example Punjabi it’s… the question actually hasn’t been asked in the way it should have been, so sometimes you get false results or it’s the other way round”*  **Not routine_not all patients need it**  *AHP7: “…our ITU is split between general and cardiac and a lot of our cardiac patients are kind of short stay, they’ll come in post-operatively but a lot of them do go to the ward quite quickly so it’s [CAM-ICU] not done as routinely on the cardiac side unless we’ve got patients who have been in longer, we do have a few longer stay patients but then it’s almost like we have to ask them to do a CAM-ICU, just because it’s not embedded routinely in practice on that side”* | **A tool differentiating between delirium types**  *AHP7: “I think there could be more maybe looking at hyper and hypoactive delirium, I don’t think it really truly differentiates hyper, hypo and mixed delirium.”* | **Long ICU stay_failed sedation holds predicts delirium**  *AHP7: “I find that a lot of the time with patients that have been in a really long time, it’s quite easy to almost predict who are going to be CAM-ICU positive, if we have patients who’ve been in kind of 10, 14 days failed multiple sedation holds”*  **Language translations**  *AHP7: “I can’t really think of anything specific. I think maybe versions in different languages would help”*  **Measure inattention_people not engaging**  *AHP7: “I probably flag concerns if I’m seeing people from a communication point of view that they’re really struggling to engage or their attention’s really fleeting and… or they can’t really engage very well with a swallowing assessment, it could be that they’re not… they’re too agitated or just yeah, not really engaging and following the commands of the assessment”* |
| **CON8**  **CAM-ICU** | **CAM-ICU every shift but not well completed**  *CON8: “I think CAM-ICU’s often not done very well.”*  **Use it because we always have**  *CON8: “It’s just what we’ve always used, I would say. It’s what we’ve used since we’ve started being more interested in delirium.”*  **Screening not viewed as valuable**  *CON8: “I think rather than just think that they [nurses] know whether a patient is delirious or not by how they’re acting – and probably question why they need to be screening at all – but they do it anyway ‘cause we ask them to.”* |  | **Question on change from baseline not well understood**  *Con8: “I think some of the questions are difficult to understand particularly the fluctuation from the baseline I think is often misunderstood. I think people often think the baseline is what’s been happening for the last couple of days rather than the patient’s actual baseline. From a screening point of view I think that’s the bit that I don’t feel the nurses always understand and that’s the bit that we have to keep going back to with education”*  **Hypoactive patients missed**  *CON8: “I think they are [sensitive enough] for hyperactive delirium and hypoactive, I’m less sure. I think that’s because people are less bothered by people who are sat in the bed not bothering.”*  **Not good when translated into other languages**  *CON8: “I think delirium screening and management of delirium is definitely harder in patients who don’t have English as their first language.”* | **Unsure- the training in the tool and applying it matters**  *CON8: “There may be, there probably are better tools. I think like most things often it’s the bundle that matters. Applying that well is sometimes better than having a better tool but now applying it well.”* |  |
| **AHP9**  **CAM-ICU** | **CAM-ICU but not well picked up**  *AHP9: “I think it’s [delirium] really badly picked up on our unit and it’s a huge thing that makes a massive difference to length of stay”*  *AHP9: “We use a ISO which is an electronic system and it’s flagged up on that that it has to be done every shift. You can’t save your flow sheet chart without having clicked delirious, not delirious, on the CAM-ICU. It is 100% done on the system. Whether it’s actually done in real life, I don’t know.”* | **Picks up delirium in obvious cases**  *AHP9: “I think again with the right sort of patient it can.”*  *AHP9: “…some cases are very obvious aren’t they if they’re completely delirious? It’s a very clear yes and no. I think the CAM-ICU is good for that but the middle ground sometimes are they Not just a little bit confused because they’ve got pre-existing early onset dementia or something? Are they covering it up really well and know what answers to say to not get the delirium tick, if that makes sense?”* | **Not sensitive**  *AHP9: “…some patients if you ask them some questions they’ll answer completely normally. Actually when we do a therapy session – and we sit them on the edge of the bed and spend an hour with them – they’re hugely delirious. It’s unless you know the patient or actually try and challenge them in a bit more of a high level way that the delirium comes out.”*  **International nurses-language barrier**  *AHP9: “I think there’s a lot of overseas nurses that English isn’t great. If you have a delirious patient that’s hard of hearing and you have got a nurse that English isn’t their first language, it’s very confusing for the patient regardless of if they’ve got delirium or not.”*  **Problematic for pts with neuro insult**  *AHP9: “If they’ve [patients] had a big neurological insult, they can’t actually understand the questions or process what we’re trying to ask them. They might not be delirious, they just can’t get their answers across or able to say yes or no.”*  **Change from baseline Qs difficult**  *AHP9: “Then ‘has the mental state changed in the last 24 hours’. If you’ve just come onto shift, you’re relying on the person before you to say, ‘oh yeah, they were fine this morning but now they’re really drowsy’ or this or this. I think that one is quite subjective, isn’t it?”* | **Different measure for longer stay pts**  *AHP9: “I think CAM-ICU does have a place in ICU. I think for specific other types of patients, a different one would be maybe more sensitive to the longer staying patients.”* | **Need to have a more in depth conversation**  *AHP9: “What I mean by that is not just ask the CAM-ICU questions but try and have a conversation with them. Looking at photos or if we know who’s in the photo and they say someone completely different”*  **Family photos**  *AHP9: “Actually if I picked a different photo from their photobook and asked them who that one was this time and then a different one in the next time”*  **Scale rather than yes/no**  *AHP9: “With a different type of screening tool – maybe from a zero to 10 or something on a scale – you can then use it to measure, oh actually the delirium’s improving or not rather than no or yes or delirium’s present, not present or whatever the answers are. Then you can actually functionally use that score, can’t you, because it’s very hard to get any data going forward using CAM-ICU, I think.”* |
| **CON10**  **CAM-ICU** | **CAM-ICU every shift but doesn’t happen**  *CON10: “…we should be performing the CAM-ICU score at start of nursing shift every day so twice a day but it doesn’t happen. Every time we audit it we’re 5% to 10% and we’ve tried all sorts of things in terms of education, reminding people to do it, making it part of the daily checks. It just doesn’t happen.”* |  | **Low confidence in CAM-ICU**  **Qs feel “silly”**  *CON10: “I think there’s very little confidence in it as a tool. Plus people feel a bit silly doing it. If you’ve got somebody who doesn’t have any delirium and you’re asking them silly questions, you feel a little bit self-conscious. Then it also seems daft to do the test in somebody who’s clearly delirious [laughs] ‘cause they don’t respond anyway.”*  **Not sensitive- don’t see value in screening**  *CON10: “Quite often you might do a CAM-ICU on a patient and it says they’ve not go delirium but then they clearly have so you’ll try and treat them anyway. […] people don’t like doing it [CAM-ICU] and don’t see the value of it.”* |  | **Use PRE-DELIRIC to risk assess those who need further screening**  *CON10: “It’s like a risk factor assessment of the patients risks of delirium. I think it’s called PRE-DELIRIC. […] I wonder if having a risk predictor [up front 31:36] come up first so that we’re not just doing it, we’re not expected to do it on 46 patients twice a day”* |
| **CON11**  **CAM-ICU** | **CAM-ICU every shift**  *CON11: “CAM ICU screening tool once per nursing shift”*  **Planned audit to check completion**  *CON11: “Well, that is the ideal, that it's done once per shift, isn't it? [I: Yeah]. We are about to roll out some trust audits which we're going to look and see how much it's actually being done once per shift. Yeah, maybe patients don't have the CAM ICU done because they appear well and they're on their way to step down to the ward.”* | **Acceptable, works well**  *CON11: “I find it an acceptable tool. I think it works. I personally feel like it works well.”*  **Easy but need training**  *CON11: “I think once you're familiar with the CAM ICU it's easy, but I think if you're unfamiliar with it it's probably a slightly complicated flowsheet to go through which is partly why we're focusing on the training”* | **Language barrier**  *CON11: “You obviously have issues with language for some patients, so communication and language can be a barrier”*  **Doesn’t work for pts too agitated or sedated**  *CON11: “I think if you've got patients who are very agitated they can't complete it with you, if they're too sedated they can't complete it with you. Yeah, I think it's a good tool.”* | **Nurses need training**  *CON11: “If the nurses aren't trained and confident with the CAM ICU tools then you run into some difficulties and we may get more patients here kind of classed as unable to be assessed”* | **No**  *CON11: “I mean, I feel it's good! So, I don't know!”* |
| **CON12 & ACCP13**  **CAM-ICU** | **RASS & CAM-ICU completed but not well understood**  *CON12: “So we are supposed to do the RASS Score and the CamICU but our feeling when we’ve audited this before is that although they’re being filled in people don’t really understand what the RASS Scores actually are”*  **Meaningless tick box exercise**  *NUR13: “I think everything could be done better. I think it is a tick box exercise. It doesn’t mean anything meaningful”* | **Picks up delirium if done correctly**  *NUR13: “I think it probably does if it’s done correctly.”* | **Time consuming and not good for pts who cant vocalise**  *NUR13: “Time consuming. I think it’s okay if you’ve got a patient who can vocalise responses. I think it becomes more difficult when people are unable to and then personally having to use the pictures. I hardly ever see anybody here who do CAM ICU. I would have doubts about how well the CAM ICU tool is used.”*  **Odd questions that normal pts fail to grasp**  *CON12: “You see patients and if they’re not delirious they look at me like I’m stupid if I say ‘can a stone float on water?’ It’s like ‘What?’ They think they’re being tricked. I also think to save a heart, I don’t think I can do that very well. [laughs]”*  *NUR13: “For the save a heart I often have to explain to the patient I’m going to do this but we’ll do a trial one first and sometimes they just can’t do it on the first go but it’s because they haven’t really twigged what it is that you’re asking them to do, even the ones that I know are completely compos mentis that have been happy to do a screening project on.”* | **Simpler**  *NUR13: “If you could make it more simplified I think that would probably work very well on the shop floor.”* | **BIS monitoring**  *CON12: “Yeah, like a BIS monitor.*  *NUR13: Yeah, I think the way that our units are going in terms of they do love a bit of tech and going forward that is the future I think.”* |
| **CON14**  **CAM-ICU** | **CAM-ICU but audit revealed its patchy**  *CON14: “the CAM-ICU is supposed to be recorded twice a day by the nurse. When we have audited it, it’s very patchy whether that’s actually done.”* | **Simple and easy to deliver**  *CON14: “It’s simple, so it’s easy to deliver. Beyond that, I’m not sure it’s particularly robust, but it is easy to deliver.”* | **Misses some patients due to fluctuating delirium**  *CON14 “I guess it could be oversimplistic. You could miss some patients, I guess, but because it’s easy and it’s a bedside test, it’s probably the best of both worlds in a way because you can deliver it. It’s not hard to deliver.”*  *CON14: “I’m sure you’re missing patients and the other thing is that the conscious state does fluctuate. It’s a snapshot of one point during the day and people will fluctuate and change during the day.”* |  | **Guided by those with special interest**  *CON14: “No. I’m usually happy to be guided by people with a specialist interest in this who will come up with something better and easier to use.”* |
| **CON15**  **CAM-ICU** | **CAM-ICU once a day but its inconsistent**  *CON15: “The screening is sometimes a little bit inconsistent, so we aim to try and screen patients every day, but that doesn't always happen. I think that's because people sometimes rely erm, thinking that it's not clinically apparent”* | **Reasonably good, picks up most pts:**  *CON15: “I think it's a reasonably good screening tool. I think it picks out the main presenting symptoms and signs of delirium”*  **SAVEAHEART Good:**  *CON15: “I think the fact that if you pass the SAVEAHAART you don’t have to progress onto the other questions, because you've excluded it from that point, that's quite a nice way of screening it, so you’ve got that easy part of doing that screening test at the beginning.”* | **CAM_ICU can be complicated and difficult for pts to understand**  *CON15: “I think people may struggling in two ways, so having the confidence to understand the test and to perform it, and secondly in patients who have difficulty communicating, how to assess it in those patients, which can be difficult.”*  **SAVEAHEART difficult for pts with muscle weakness**  *CON15: “so Guillain-Barré Syndrome, we've had a couple of patients who have been difficult to communicate because of muscle weakness.”*  **Fluctuation doesn’t happen**  *CON15: “I think the fluctuation in conscious level doesn't always exist, so that fluctuation sometimes there isn't that fluctuation, but I know that's one of the criteria for it.”*  **Not easiest test to do:**  *CON15: “I think it's not always the easiest test to do. I think that might put nurses off doing it.”* | **Miss hypoactive pts**  *CON15: “I think it's just people might focus on those patients who are presenting quite obviously clinically with delirium, to assess those patients, but we probably miss patients by not screening everyone routinely.”*  **Need to be familiar and confident with the tool**  *CON15: “…you've got the scoring test, so I think being confident and familiar with a screening tool is probably the key skill in terms of identification.”* | **Indicators of delirium: Pt not progressing/ inattention and poor/ reversed sleep**  *CON15: “soft indicators […] not weaning well, not progressing with their treatment […] inattention, that's a big one, not being able to focus and follow a conversation, or follow commands […] repetitive movements. […] absence of a good day-night's sleep cycle”*  **Simpler tool:**  *CON15: “I guess for example, we've been doing GCS scores forever, and then they brought out AVPU scoring in recent times, and that's used more by nursing staff”*  **Observable behavioural element:**  *CON15: “I think there could be an observe behavioural kind of element to it as well”*  **Identify those not engaging in rehab:**  *CON15 “You sometimes find it’s labelled as ‘The patient is depressed,’ or ‘This patient’s fed-up, and doesn’t want to engage with rehab, or want to do things,’ when on closer examination and talking to the patient you might find out that they’re having delirious thoughts about things, aren’t sleeping well, and all those characteristic things that promote hyperactive delirium.”* |
| **NUR16**  **ICDS** | **Pre-operatively asses pts risk of delirium:**  *NUR16: “Pre-op electives we have a structured booking system, and part of that is we gather that past medical history, so then we would alert the consultant group, the pharmacist, that there's a patient coming in who's got a high risk of delirium, particularly if it's an alcohol excess, or a drug problem, then at least they can preemptively start to prescribed things or be aware that there's going to be potential issues.”* | **Quick**  *NUR16: “It's really quick, takes about seconds. It's all digital, so you tick the boxes, and the score adds up at the bottom, really quick.”* | **Need action plan early on**  *NUR16: “I think they [nurses] may know they have to fill it in, but I don’t know if they then have the skills to know what to do with it. I think they often let it get to the point where somebody is triggering really high, and then it leads to chemical restraint, rather than putting in an action plan before.”*  **Hypoactive not well understood**  *NUR16: “I think probably the hypo one people struggle with the psychomotor slowing, people will be like, ‘Oh, what is that?’”* | **Satisfied with ICDS**  *NUR16: “I think we're satisfied with it; we've used it for a long time. It needs things around it like any screening, it needs the extra plans, it need the support documents, but as a recognition yeah, it's fine.”*  **Qs- Do you feel safe?- pick up hypo pts**  *NUR16: “…hypo. […] people struggle with that, because they think, ‘Oh, my patient’s very compliant and quiet and not saying anything, but actually they’re totally terrified inside […] we’ve introduced at the minute is a question to the patient, ‘Do you feel safe?’ to open up that conversation”* | **Subtle signs- wild eyes** *NUR16: “But then there's also the physical signs that you would look for around inattentiveness, the wild eyes that we always see happen in the paranoia. There's often very subtle signs that somebody's going to tip into delirium, and we have guidance around predicting patients who are likely to become delirious. We know that the case mix that's likely to have problems, where they’re post-elective or post-emergency.”*  ***Experienced nurse knows signs_paranoia***  *NUR16: “ …that just comes with experience to know that somebody is going to tip over that night or in the day, it’s looking at their eyes. You can often see that they're about to blow big time. But junior staff won't recognise that, and that as soon as a patient starts with, ‘What are you doing, what are you putting in my drink?’ ‘Where’s that…?’ ‘Where’s my family, why are you keeping them?’ You think, ‘Oh, paranoia is coming along.’”* |
| **NUR17**  **CAM-ICU** |  | ***Good:***  *NUR17: “It’s good that it’s a flow chart, you can use it.”* | **Communication issues when using the tool**  *NUR17: “I find the problems with it really are the fact that there can be communication issues, the patient might not hear you, they might be in such a state of anxiety and fear that they don’t really understand what you’re saying because they can’t comprehend it at the time. They might not be delirious but they might be so frightened that they don’t really understand that.”*  **Not screening lightly sedated pts:**  *NUR17: “and a lot of people were putting that they couldn’t assess them at -3, or that they would only do the screening once they’re off the sedation, they’re awake. Quite a few thought that when you would do a delirium screening.”*  **Hypoactive missed:**  *NUR17: “The other thing was, not everybody recognised hypoactive delirium, and that is so important, that one is so detrimental to the patient and it can give worse outcomes, because it goes undetected”*  **Subjective:**  *NUR17: “one person might do it and deem them as CAM positive, another person might do it and deem them as CAM negative, and that does happen. And then patients who are hard of hearing or there’s a communication issue between maybe the nurse and the patient, I think that’s a problem with it.”* | **YES- include pain and facial expressions**  *NUR17: “I do. I do, yeah. And maybe it could be maybe a mixture of this, combined with a pain tool which takes into account things like facial expressions, you know, whether they’re grimacing”*  **Do they feel SAFE**  *NUR17: “if you’re in a state of fear in the flight or fight mode, you’re not going to listen to silly questions – not silly questions, but you know, they just want to know that they’re safe I think.”* | **Need to look at ROOT cause of agitated behaviour:**  *NUR17: “if somebody’s agitated they might be deemed as CAM positive when actually they’re not. They might be in a lot of pain, they might be absolutely terrified, they might want the toilet – that’s a big thing with our patients, they might just want the toilet and they can’t get that communicated”*  *NUR17: “…a couple of nights ago we had a patient and he’s got a tracheostomy and he has suffered with intermittent delirium, but he was classed as delirious during the night shift and our delirium policy includes the use of restraints, they’re just soft cuffs that we place on the patient […] I came on the morning shift and he said, ‘I just wanted the toilet.’”* |
| **NUR18**  **CAM-ICU** | **CAM-ICU Every 12 hours**  *NUR18: “So we got the CAM ICU put on to Nerve Centre and then the nursing staff get an alert every 12 hours to complete the CAM ICU in a digital format.”*  **Screening ‘tickbox’ no treatment so doesn’t lead to change**  *NUR18: “Because really all the treatment is the same as the prevention which you’re already doing, so I think that’s sometimes a barrier to kind of doing it [screening], because it’s like whether people see the point of it.”* | **Computerised protocol**  *NUR18: “So we got the CAM ICU put on to Nerve Centre and then the nursing staff get an alert every 12 hours to complete the CAM ICU in a digital format.*  *I: Have you noticed a difference since doing that?*  *NUR18: Yeah compliance in kind of doing an assessment is better”*  *NUR18: “I think having this digital solution that kind of forces people to do it every 12 hours has made it a lot easier with compliance to do it.”* | **Baseline Qs misunderstood- need for more training**  *NUR18: Yeah compliance in kind of doing an assessment is better, but I’m not sure about the quality of the assessments all the time, sometimes – the key question of has there been a difference from someone’s baseline, often people just say no, there hasn’t been a change. Then that means you don’t have to do the rest of the screening. So it is easy isn’t it?*  *I think there’s a need for a bit more training because, so for example if someone in – it’s hard and they can’t speak, it’s hard to tell if there’s a difference from their baseline isn’t there? So I’ve been encouraging staff to kind of move beyond that question and to go to the next one, to then be a bit more robust of a screen.”*  **Not sensitive:**  *NUR18: “How confident? Probably more confident in diagnosing it, I think the CAM ICU doesn’t always marry up with what you’re looking at. Sometimes you can look at someone or interact with them and think they’re delirious, but then according to the CAM ICU they’re not. So that’s difficult because you then question I suppose your clinical judgement or it kind of means that you don’t have faith in the screening tool, which means people are less likely to do it.”*  **CAMICU Awkward:**  *NUR18: “It’s quite odd isn’t it to ask them to do that task if someone is awake and alert and talking to you? So maybe the kind of awkwardness of addressing that and having to say to them, “We’re doing this test in case you’re delirious,” then you’d have to explain what delirium is. Sometimes I wonder if that sometimes stops people, that sense of awkwardness.”* |  |  |
| **NUR19**  **CAM-ICU** | **Ad hoc. Hyperactive picked up more than hypoactive delirium**  *NUR19: “I think a lot of it is probably ad hoc if I’m honest. Usually from patients more being the hyper delirium as opposed to the hypo delirium.”* | **Picks up delirium:**  *“NUR19: I think I’m quite confident, I think I’d probably say about sort of 80%, I think the ones it doesn’t pick up on are those where it might appear to be delirium but it might be something else. Equally, I think if you’re then flagging up it’s delirium and the consultant feels it’s not then at least it’s been flagged up.”* | **New nurses not confident:**  *NUR19: “I would say a good 80% are probably not confident, we’ve had quite a new [Inaudible 0:06:33] of Band 5’s as well who are probably – of everything they have to learn it’s probably way down the list of things that they have to learn. That will take time to build up.”*  ***Confusing:***  *NUR19: I think it can be confusing if you’re not really sure what you’re doing or you don’t remember the steps, I think there are a lot of steps. I think it’s very linked with sedation and sedation scoring and so in order to understand delirium you also have to understand sedation and do the sedation scoring as well first.*  *So I think that bit is probably not very understood”*  **Not sensitive- need to look at pt over a few days:**  *“NUR19: I think hypo active delirium is probably – well I think the, kind of, observation over a longer period of time, so we obviously tend to do shift by shift but actually if you look at somebody over two or three days and you see periods of confusion versus periods of just not doing anything. Family saying they just don’t seem to be making sense, fidgeting, agitated, picky at certain times, I think what tends to happen is you don’t pull it all together. So you get a snapshot at a certain period of time when you do the CAM ICU and they’re behaving perfectly normally and then it’s, “Oh they’re not delirious.”*  **Too long**  *NUR19: “So yeah, I think it probably, there is an assumption it takes too long, or it takes too long to even find the questions, so we’re not even going to bother with the screening.”*  **Not easily accessible:**  *NUR19: “At that point we sign post them to where – so on our electronic patient record the questions are there, the problem is where to find the screening is quite convoluted, so it’s actually getting to it in the first place.”* | **Easy and quick**  *NUR19: “So whilst I like CAM ICU it’s – there’s a lot of thought process that has to go into it to get to a score and if I’m honest, trying to even get a bedside nurse to do it once a shift is quite hard going.”* |  |
| **CON20**  **CAM-ICU** | **CAM-ICU not always completed as busy:**  *CON20: “But as I mentioned, there are a few nurses that don’t do this routinely because probably of their age on ICU. They’re very junior. They don’t know how to use because they’re busy with other things. So sometimes.”*  **No protocol:**  *CON20: “We do not have a policy on delirium on our critical care unit. That might be the reason that they don’t feel under compulsion to do this test. So, some of them they might not be knowing about CAM ICU or this screening tool, and that’s why they’re reluctant or they forget or they don’t find the time during the day time to go through this tool.”* | **Good:**  *CON20: “I think it’s a good tool to assess whether the patient is confused or whether they are disorientated. So, I think it’s good.”* | **CAM-ICU- difficult on semi-conscious patients:**  *CON20: “It is only for the conscious patients and those that can respond. But those patients who are semi-conscious, who cannot see, who cannot respond, then that’s difficult on them to use.”* |  |  |
| **CON21**  **CAM-ICU** | **Ad hoc screening:**  *CON21: “I would say that our policy says we do CAM-ICU checks which is nurse led once a shift, so twice a day. In reality it’s probably a bit more ad hoc, a CAM-ICU will be done when we feel there might be an indication that there’s some delirium.”*  **Not agitated- no time- won’t do an assessment:**  *CON21: “I suppose possibly the resource of time. We will do a focussed assessment on the things that matter. So we don’t feel there is a problem, we don’t feel the patient is agitated, then we won’t do a CAM-ICU. And that’s possibly a time issue in the same if we don’t think there’s a neurological problem, we won’t do a neurological examination of the patient.”*  **Not confident in diagnosing and screening:**  *CON21: “I’m less confident now because I’ve realised it’s a lot more complex than I perhaps thought it was.”* | **Well validated but not the easiest:**  *CON21: “as I understand it’s quite well validated, I think the evidence base says it’s probably the best tool we’ve got at the moment. […]But equally it’s not the easiest and I usually have to get out my little crib sheet every time I do it just to make sure I’m getting the questions right.”*  **Staff- level 3 care:**  *CON21: “But we’ve got an increase in our nursing workforce with, what are they called, associate nurses and then we’ve still got our HCA’s. So we try to where a patient does have a degree of delirium, particularly hyperactive delirium, then we might place an extra pair of hands at that bedside to try and help keep the patient safe without just sedating them. Environmental things, again so noise is awful but light yes we try and keep it light during the day and dark at night. And certainly when I do my evening ward rounds I’ll instruct the doctors to not do a review of that patient who’s trying to sleep, don’t wake them up just to check everything’s still okay. “*  ***Accurate- better than clinical judgement:***  *CON21: “there’s been a couple of times I’ve been quite surprised the patient is delirious but then actually they’ve got quite good attention and they can do the [12:02 inaudible] questions quite well. So it does sometimes surprise me and then I – maybe that’s my clinical judgement is wrong and the CAM – I would say the CAM-ICU is correct. “*  *CON21: “sometimes I’ve seen the most distress, they’ll assume that they’re delirious and then when you do a closer inspection and a CAM-ICU and talk to them and then talk to them in a bit more detail. I realise that actually they’ve got good attention, they don’t have that acute delirium and they’re just really anxious and they’re trying to climb out of bed because they genuinely fear for their life, even though they fully understand what’s going on.”* |  | **No**  **Need a care package to follow the screening tool:**  *CON21: “I mean I still come back to the point that even if I do it and they – it confirms or refutes my opinion, I’m still left with not a lot of intervention to actually make a difference. So I don’t think it’s the tool itself that’s stopping me using it regularly, it’s the problem of managing delirium that stops me using regularly.”* |  |
